# Ancillary tests for brain death

**DOI:** 10.3389/fneur.2024.1491263

**Published:** 2025-01-07

**Authors:** Shyam Duvuru, Vivek Sanker, Rajeeb Kumar Mishra, Arvind K. Sharma, Shir Lynn Lim, Nisha Baskar, Vijay K. Sharma

**Affiliations:** ^1^Apollo Specialty Hospital, Madurai, India; ^2^National Institute of Mental Health and Neurosciences (NIMHANS), Bangalore, India; ^3^Zydus Hospitals, Ahmedabad, India; ^4^Yong Loo Lin School of Medicine, National University of Singapore, Singapore, Singapore

**Keywords:** brain death, ancillary tests, death by neurologic criteria, brainstem functions, cerebral circulatory arrest, transcranial Doppler

## Abstract

**Background:**

Ancillary tests are often used in the determination of death by neurologic criteria (DNC), especially when the clinical examination is inconclusive. DNC is widely accepted, as defined by the comprehensive report of the World Brain Death Project. However, there are several medical, legal, religious, ethical, and social controversies. Accordingly, “premature” and “delayed” diagnoses of brain death attract these issues.

**Methods:**

Depending upon the availability and experience of the managing medical teams, various ancillary tests are employed for an early and supplementary diagnosis of brain death.

**Results:**

We describe the practicality, test performance, and utility of some of the commonly employed ancillary tests for the diagnosis of brain death in clinical practice, along with their case examples.

**Conclusion:**

Brain death is a clinical diagnosis determined by history, physical examination, and adherence to recommended criteria. All ancillary investigations are used as supplementary tests with variable accuracy parameters. These ancillary tests often facilitate an early and “timely” diagnosis of brain death.

## Introduction

Death by neurologic criteria (DNC) was first described by French neurologists in 1959, who referred to it as *le coma dépassé* (beyond coma or irretrievable coma) (1). This concept gained widespread attention in 1968 when the formal standards for its determination were established, and the term “brain death” was coined (2).

Ancillary tests are often used in the determination of DNC, especially when the clinical examination is inconclusive. DNC is widely accepted, as defined by the comprehensive report of the World Brain Death Project (3). However, there are several medical, legal, religious, ethical, and social controversies. In particular, the issues surrounding “premature” and “delayed” diagnoses of brain death attract these issues.

The majority of centers make all efforts to ensure a timely diagnosis of brain death as well as avoiding a “premature” or “very late” diagnosis. From a social and family point of view, an accurate determination of brain death is essential for providing closure to relatives and for discontinuing somatic mechanical support in the deceased individual.

In general, brain death is the death of the individual due to irreversible loss of function of the entire brain. In legal terms, it is known as “DNC,” which is accepted as legal death in most jurisdictions, as determined by one or more medical professionals through the application of accepted medical standards (3). As per the recommendations laid by the World Brain Death Project 2020, prior to evaluating a patient for brain death/DNC, the patient should have an established neurologic diagnosis of complete and irreversible loss of all brain function (3). The recommendations emphasize excluding all confounding conditions mimicking brain death. The lists of confounders usually encountered are the presence of demyelinating polyneuropathy (Guillain-Barré syndrome), botulism, the presence of severe metabolic derangements, toxicities, sedative medications, hypothermia, and hemodynamic perturbations (4). The essential prerequisite for DNC is the reliable clinical examination showing permanent cessation of consciousness (coma) and loss of brainstem reflexes, including central apnea as shown by an apnea test (3). Thus, these three criteria should be clearly demonstrated clinically. The patient should be in a profound state of unarousability with maximal external stimuli and completely unaware of the surroundings consistent with the definition of coma. All the brainstem reflexes such as pupillary reflex to light, corneal reflex, oculocephalic, oculovestibular, gag, and cough reflex to deep tracheal suctioning should be absent. It is noteworthy to mention that spinal cord reflexes may still be present in patients diagnosed with brain death.

Apnea is one of the cardinal signs of brain death. It is important to highlight that the apnea test is not an optional portion of determining DNC. During the apnea test, the serum carbon dioxide increases, and the central nervous system pH decreases to levels needed for maximal stimulation of the respiratory centers in a functioning medulla. Patients with no medullary function will not be able to initiate any respiratory effort despite profound hypercarbia and acidosis. Accordingly, if no spontaneous respiratory efforts are observed after a rise of PaCO_2_ >20 mm of Hg from the baseline or an absolute increase of PaCO_2_ >60 mm of Hg at the end of the test, the test is positive for absent brainstem function compatible with brain death (4). The recent guidelines by the American Academy of Neurology guidelines further expanded the requirements of the apnea test by including the arterial pH value of <7.3 in patients who are not known to have chronic hypercarbia. Furthermore, apnea testing should be aborted if systolic blood pressure is <100 mm Hg or mean arterial pressure is <75 mmHg, despite titration of vasopressors, inotropes, and/or intravenous fluids, in addition to those patients where a progressive decrease in oxygen saturation (<85%), cardiac arrhythmia with hemodynamic instability (5). However, apnea testing may increase the intracranial pressure in some cases. It is recommended that the apnea test should be conducted after other clinical evaluations have been completed. It is important to ensure that the patient is not having any spontaneous respirations when the ventilator is set on a spontaneous breathing mode in a normocarbic state and the body temperature should be at least 36°C. Furthermore, a positive apnea test may be confounded by the high cervical spine injury and should be interpreted with caution. Finally, in hemodynamically stable patients where targeted carbon dioxide levels cannot be reached, the apnea test should be repeated after reestablishing preoxygenation, normocapnea, and a normal pH, in addition to extending the duration of the test.

According to the current recommendations, brain death should be diagnosed using “DNC.” We believe that the term “brain death” should be replaced by DNC, as the former might be construed as just permanent damage of an organ rather than death of the individual. The American Academy of Neurology recognizes that the fundamental concept underlying the accurate determination of brain death is the irreversibility of injury to the cerebral hemispheres and brainstem (5). However, in real clinical practice, many confounders may render the clinical examination unreliable, such as drug intoxication, high cervical spinal cord injury, and the use of various sedative and paralyzing agents. Furthermore, in patients with unstable cardiopulmonary status, performing apnea testing may not be considered safe. While in some cases, conducting the apnea test becomes difficult because of patients’ underlying poor oxygenation and hemodynamic instability, clinical examination remains incomplete in others such as in those with skull base fractures with extra-ocular muscle entrapments, anophthalmia, ruptured tympanic membranes, and in those with predominantly posterior fossa pathologies. In such circumstances, various ancillary tests may be employed to support the diagnosis of brain death. Accordingly, ancillary testing may be performed to assist in brain death/DNC determination if the apnea testing cannot be completed, or the findings cannot be interpreted adequately. Interestingly, in certain jurisdictions, ancillary tests are also compulsory to confirm DNC, even in patients with reliable clinical examinations (6). In general, brain death determination is fundamentally a clinical assessment, and the ancillary tests serve as surrogate means of assessment when requisite components of the clinical brain death evaluation cannot be adequately performed or interpreted (7, 8).

The commonly employed ancillary tests are aimed at assessing brain structure (computed tomography—CT, magnetic resonance imaging—MRI) or surrogates of brain function such as evaluating cerebral blood flow (digital subtraction angiography—DSA, computed tomography angiography—CTA, and transcranial Doppler—TCD), cerebral perfusion (CT perfusion, single photon emission computed tomography—SPECT), or neurophysiological function (electroencephalogram—EEG and evoked potentials) (9). According to the American Academy of Neurology guidelines, DSA, SPECT, and TCD in adults are acceptable ancillary tests, while EEG, evoked potentials, CT angiography, and MR angiography are considered unacceptable ancillary tests (5). A recent meta-analysis evaluated the diagnostic accuracy of various ancillary tests in studies that included both clinically diagnosed DNC and those with clinically suspected criteria. The analysis found a high risk of bias and proposed the need for high-quality research to provide accurate and valid measures of these tests’ diagnostic accuracy (10). While brain death determination is fundamentally a clinical assessment, the ancillary tests serve as surrogate means of assessment when requisite components of the clinical brain death evaluation cannot be adequately performed or interpreted (9, 10). We wish to make it clear that not all guidelines or consensus statements agree on the utility of various ancillary tests. Accordingly, considerable heterogeneity may be observed in the use of these ancillary tests, largely related to various factors including underlying medical conditions, clinicians’ preferences, test availability, and local expertise. We present a brief overview of the test performance and utility of various ancillary tests performed for the assessment of brain death in adults in routine clinical practice, along with relevant case examples. We wish to reiterate that our review does not refer to children and infants, primarily because of the “low to very low” quality of evidence, with the exception of radionuclide dynamic flow studies where the quality was just moderate (11).

## Single photon emission computed tomography

**Figure 1:**
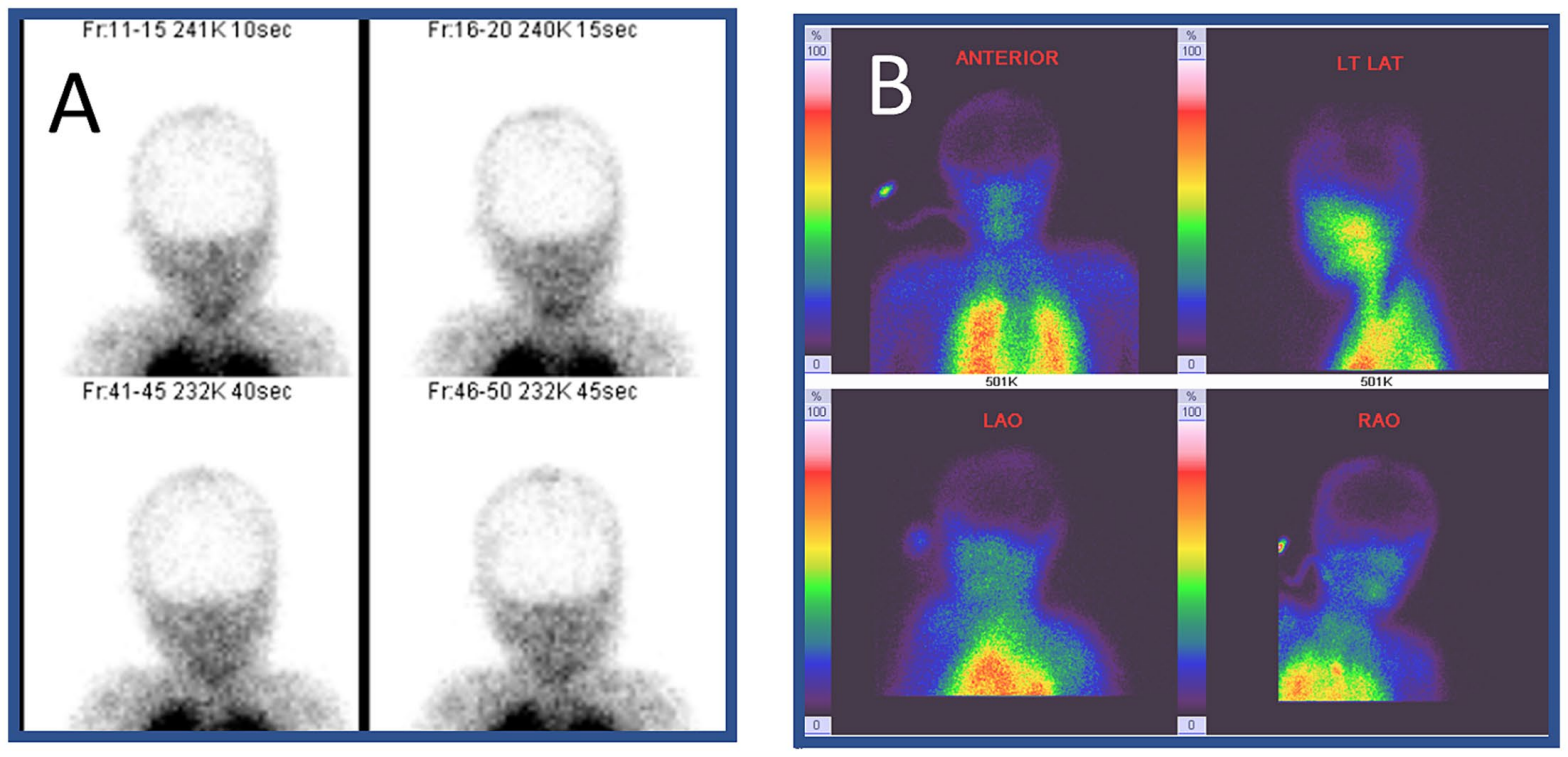
Radionuclide imaging of brain death. This 9-year-old girl suffered from severe pneumonia, requiring prolonged ventilation and extracorporeal membrane oxygenation. However, her condition continued to deteriorate. No brainstem reflexes were observed on day 7. She underwent single photon emission computed tomography (SPECT) with ^99^Technitium HMPAO. No radionuclide uptake (metabolic perfusion) was noted in the brain (hollow-skull sign) in the images in panels **(A,B)**. **(B)** Shows increased radionuclide uptake in the extracranial structures including the nose area (hot nose sign).

SPECT has the advantage of providing information about brain metabolism and blood flow, often called metabolic perfusion. In brain death, SPECT typically demonstrates no radionuclide uptake in the brain and cerebellum (the “hollow-skull” sign) (Figure 1). The “hot nose” sign, described as increased uptake in the nasal area, with no uptake in the intracranial arteries, has also been described in patients with brain death (12, 13). However, the “hot nose” sign should be used with caution for the diagnosis of brain death since it may be seen in patients with other intracranial pathologies associated with increased intracranial pressure, such as ischemic stroke, subdural hematoma, and hepatic encephalopathy (14). In addition, in some patients, SPECT may demonstrate a preserved cerebellar perfusion without cerebral perfusion and vice-versa (15). Accordingly, the reported sensitivity and specificity of SPECT for the diagnosis of brain death are 88.4 and 100%, respectively (15).

## Digital subtraction angiography

With an increasing number of acute neurointerventions, DSA has become widely available and considered the gold standard for the evaluation of intracranial flow. The sensitivity and specificity of DSA for the diagnosis of brain death are 100% (16). For brain death assessment with DSA, the radiocontrast agent should be injected into the aortic arch under pressure and should reach the anterior and posterior circulations. The results are suggestive of brain death when there is no intracerebral contrast filling, including the arterial flow at the level of entry of the carotid and vertebral arteries into the skull, and no venous drainage (17, 18). The injected contrast is often seen rushing into the external carotid circulation (19). However, DSA is invasive and time-consuming and carries a risk of contrast-induced renal injury in potential organ donors (20). In addition, some proximal opacification of the intracranial arteries due to stasis filling can be seen in DSA in brain-dead patients (19–21). In addition, clinicians should be cautious of the false-positive results in patients with hypotension and false-negative results in patients who have undergone decompressive craniectomy (22).

## Transcranial Doppler

TCD is a rapid, reliable, non-invasive, and portable test that provides real-time information about cerebral hemodynamics. It is important to note that TCD helps in establishing the diagnosis of cerebral circulatory arrest (CCA), which may be considered a marker of brain death. Since the hemodynamic changes of CCA evolve over time, the longitudinal changes observed on TCD serve as reliable markers of brain death. Compared to DSA, TCD has a sensitivity of 90% and specificity of 98% (23). However, sonographers should be careful since TCD Doppler spectra may be affected by intracranial pressure and may continue to show some residual parenchymal flow, even at a very late stage in some cases. TCD has not been validated in the pediatric population and therefore should not be used as an ancillary test in children.

In 1998, the World Federation of Neurology published a consensus opinion on the diagnosis of cerebral circulatory arrest using Doppler-sonography (24). The prerequisites for using TCD included the establishment of the cause of coma and exclusion of conditions such as intoxication, hypothermia, hypotension, and metabolic disorders. Accordingly, systolic spikes of less than 200 ms duration and peak systolic velocity of less than 50 cm/s should be recorded in both intracranial and extracranial arteries. In addition, conditions increasing the ICP should be excluded.

### TCD protocol for CCA

The recommended scanning protocol for suspected CCA involves the following steps:

- Document arterial blood pressure at the time of TCD examination.
- Use maximum power output on the TCD machine.
- Ensure that the patient has sufficient temporal acoustic windows.
- Obtain similar abnormal TCD spectra from at least 3 intracranial arteries, preferably both middle cerebral arteries (depth 50–60 mm) and basilar artery (80–90 mm).
- Exercise caution when interpreting the high-resistance flow spectra (with minimal or no diastolic flow) or reverberating patterns, especially if increased ICP is suspected. If the patient is not on ICP monitoring, repeat the test after 12–24 h to observe the expected evolution of TCD findings.

### How to interpret TCD findings in CCA?

**Figure 2:**
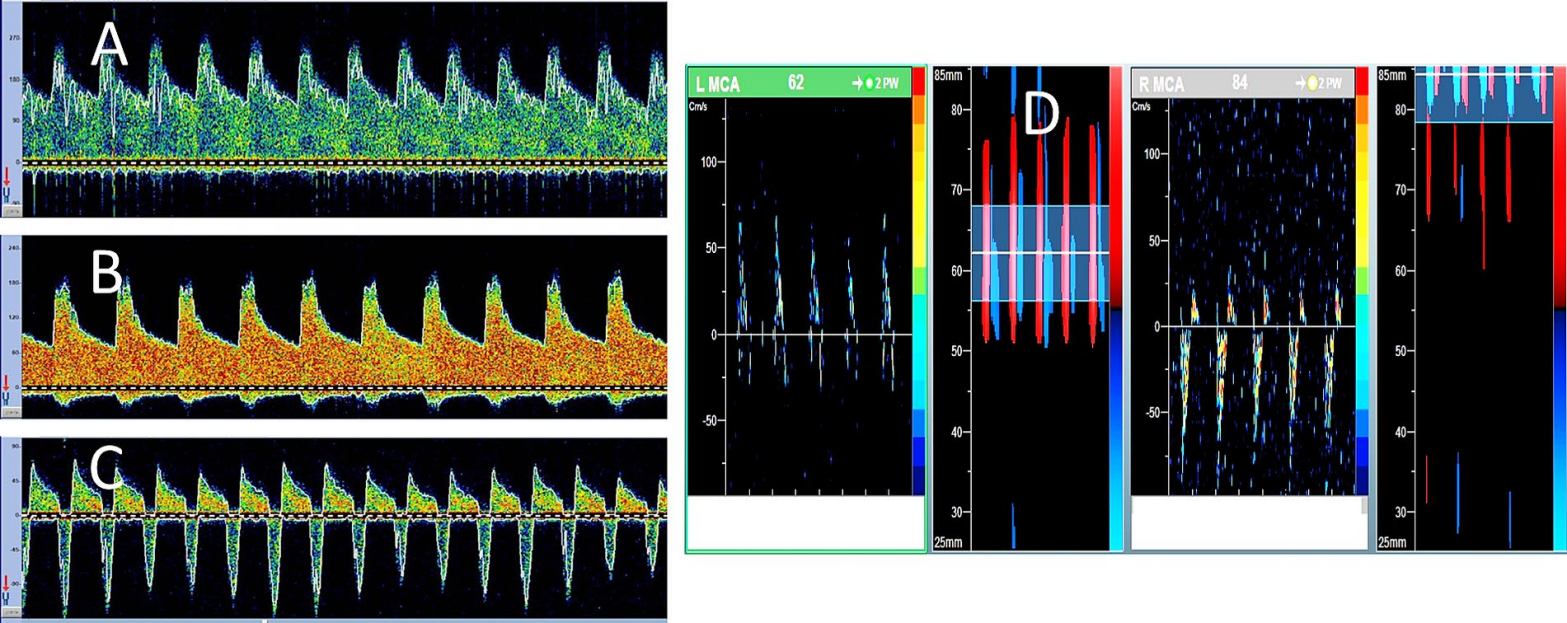
Serial transcranial Doppler (TCD) findings in brain death. A 48-year-old woman was admitted with severe subarachnoid hemorrhage due to an anterior communicating artery aneurysm rupture. She underwent urgent clipping and was monitored regularly with TCD. Her level of consciousness remained poor, while ICP continued to increase despite all therapeutic measures. During the 7 days of follow-up, his TCD spectra showed serial evolution from a normal-looking waveform **(A)** on day 2 to a higher resistance waveform **(B)** on day 4 to the oscillating pattern (suggestive of cerebral circulatory arrest) on day 5 **(C)**. No brainstem reflexes were observed, and TCD spectra showed further evolution of the oscillating Doppler spectra in both middle cerebral arteries **(D)**.

- Positive MCA or BA end-diastolic flow: No cerebral circulatory arrest.
- Absent end-diastolic flow: Uncertain cerebral circulatory arrest (either too early or too late to diagnose).
- Reversed minimal end-diastolic flow: Possible cerebral circulatory arrest (continue monitoring and ensure diastolic BP ≥50 mmHg).
- Reverberating flow: Probable cerebral circulatory arrest (confirm in both MCAs and BA; monitor for 30 min if TCD is the sole ancillary test). TCD findings in a patient with CCA are shown in Figure 2.
- Late stages: No detectable flow signals may be observed on TCD.

## Electroencephalography and related electrophysiological tests

The reliability of EEG to confirm brain death remains a controversial subject (25). While an electrocerebral silence on EEG may be suggestive of brain death, it is regarded as an infrequent occurrence. According to the World Brain Death Project, it is suggested that EEG is no longer needed as a routine ancillary test in adults due to its inability to assess the function of deep brain structures and brainstem function (3, 4).

**Figure 3:**
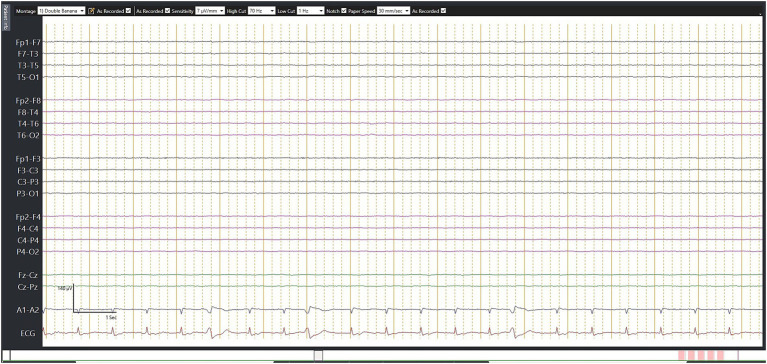
Electroencephalography (EEG) in brain death. This 67-year-old man suffered from a fracture of his right hip following a road traffic accident. He underwent successful surgery. However, on day 7, he suddenly collapsed during rehabilitation, requiring prolonged cardiopulmonary resuscitation. He was noted to have a massive pulmonary embolism, which was treated with thrombolysis. However, his neurologic status continued to deteriorate. Although this EEG was not performed according to the recommended brain death protocol, no detectable electrical activity observed during 30 min of EEG recording may still serve as supplementary criteria.

Furthermore, if performed as an ancillary test, EEG should be used in conjunction with somatosensory and brainstem auditory evoked potentials given the limitations of EEG for evaluating brainstem function. It is recommended that if EEG is performed, it should be interpreted in accordance with the recommended criteria (26). The American Clinical Neurophysiology Society has laid down several criteria to determine electrocerebral inactivity (ECI) using EEG (26). The guidelines define ECI as the absence of electrical activity over 2 μV from scalp electrodes with an interelectrode distance of at least 10 cm for at least 30 min of recording (Figure 3). To accurately demonstrate brain death by EEG, the guidelines recommend complying with the outlined standards for the EEG recordings (26). While EEG is readily available, it is limited to only assessing cortical function and is confounded by electromagnetic environmental noise, sedation, hypothermia, toxic states, and metabolic disorders. The reported sensitivity and specificity of EEG in brain death are 53–80 and 97%, respectively (25).

Somatosensory evoked, visual evoked, and brainstem auditory evoked potentials are other bedside electrophysiological techniques, which have been used as ancillary tests for an early diagnosis of brain death. They are totally dependent on the integrity of their respective neuronal pathways and may be affected by sedation. They have limited specificity and are not used in isolation in the evaluation of brain death (27).

## CT and MRI of the brain

**Figure 4:**
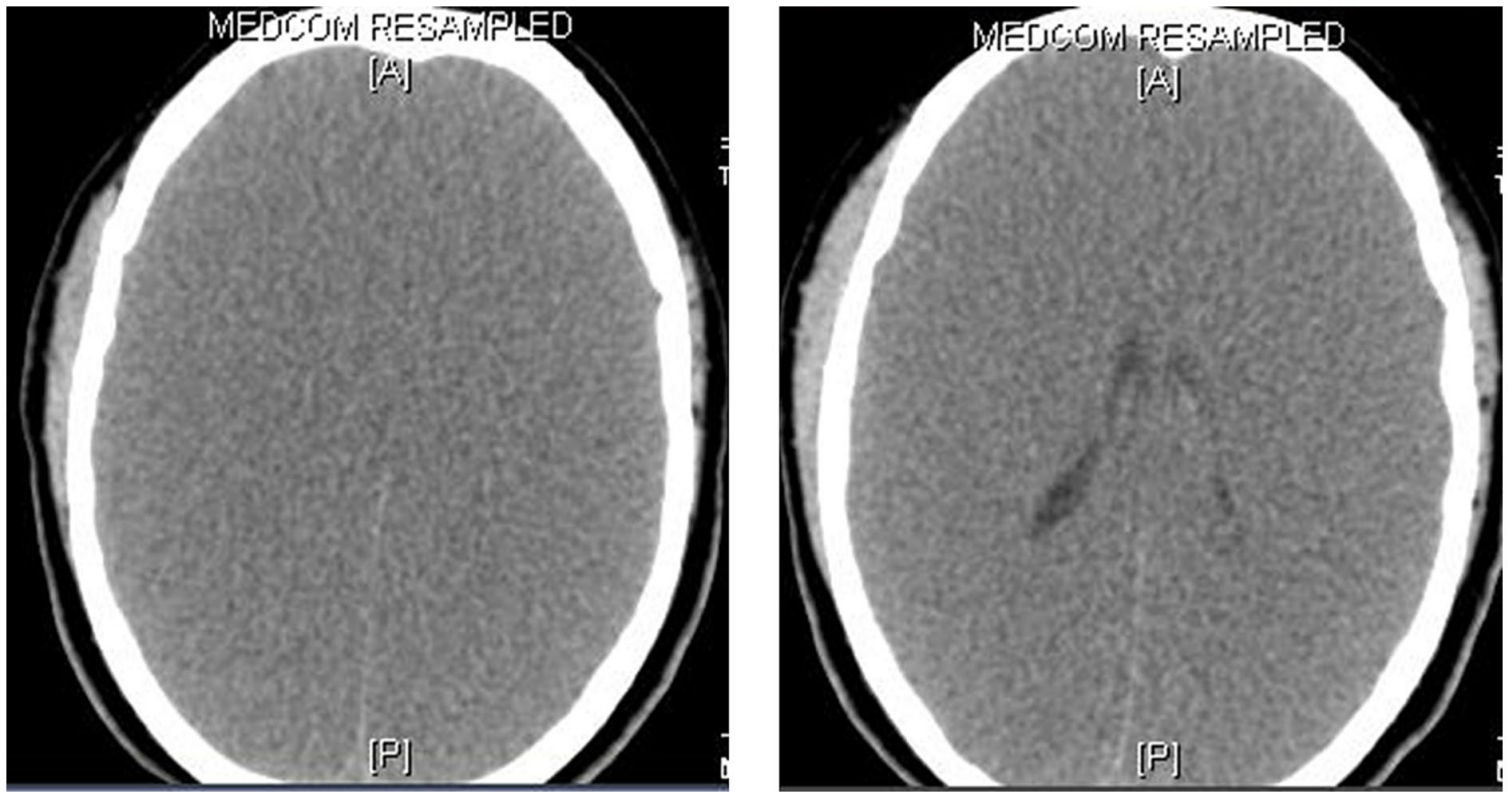
CT scan of the brain in brain death. This 8-year-old boy presented with a rapidly deteriorating level of consciousness and generalized seizures for 2 days. He was diagnosed with viral encephalitis. While on mechanical ventilation, his pupils were noted to be fixed and dilated on day 4 and there were no elicitable brainstem reflexes. The CT scan of the brain showed generalized cerebral edema, loss of the gray-white matter junctions, and effacement of the sulci, ventricles, and basal cisterns.

**Figure 5:**
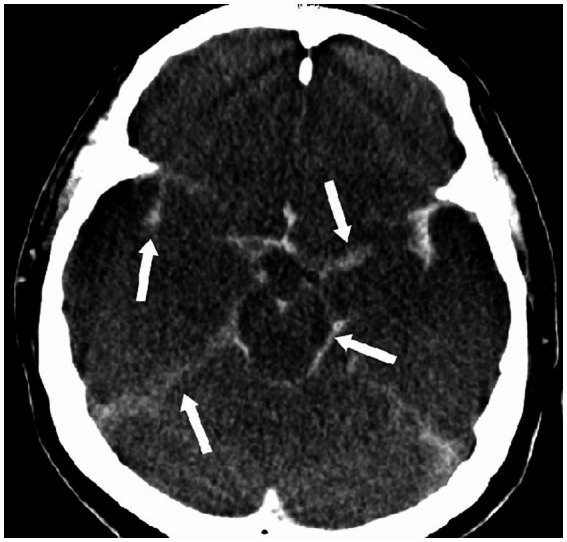
Pseudosubarachnoid hemorrhage. This 47-year-old man suffered from sudden cardiac arrest in a supermarket. Cardiopulmonary resuscitation was initiated by his friends until the arrival of paramedical professionals. Initially noted ventricular fibrillation was successfully cardioverted by two electric shocks, and he was intubated and brought to our emergency room (downtime 36 min). No brainstem reflexes were observed. The CT scan of the brain showed increased attenuation within the basal cisterns that mimics a subarachnoid hemorrhage.

CT and MRI are widely available, and either of them is performed in patients with brain injury, altered level of consciousness, or brain death (28). Being faster and relatively cheaper, CT is often the initial imaging modality (20). While both CT and MRI provide reliable information about the extent of cerebral damage and edema, the latter provides more detailed information, especially the early changes of hypoxic-ischemic injury, such as loss of the gray-white differentiation and sulcal effacement (Figure 4). Pseudosubarachnoid hemorrhage has been a well-described phenomenon on CT scans of the brain, which occurs due to hyperdense veins in effaced sulci (Figure 5). With increasing cerebral edema and intracranial pressure, features of brain herniation (such as subfalcine, uncal, and through the foramen magnum) may be seen in neuroimaging studies.

We wish to reiterate that MRI provides more detailed information about the initial cerebral damage and its secondary effects. Some of these findings on MRI are the absence of intracranial flow voids in major arteries and veins and restricted diffusion due to cytotoxic edema. Additional findings include prominent hypointense signals in medullary veins due to venous stasis or venous dilatation in response to the release of substances such as adenosine after neuronal death (29). Furthermore, gadolinium-enhanced MRI can demonstrate intense enhancement around the nose and scalp (supplied by an external carotid artery) without any enhancement of intracranial structures (supplied by an internal carotid artery) (30). However, MRI is more time-consuming and is often difficult to perform in critically ill patients.

It is important to note that imaging findings on CT and RI cannot be used to diagnose brain death in the absence of clinical evidence. This information is primarily anatomical and requires the expert judgment of the interpreting neurologist and radiologist to determine whether these findings are consistent with the possibility of meaningful neurologic recovery.

## CT angiography and MR angiography

**Figure 6:**
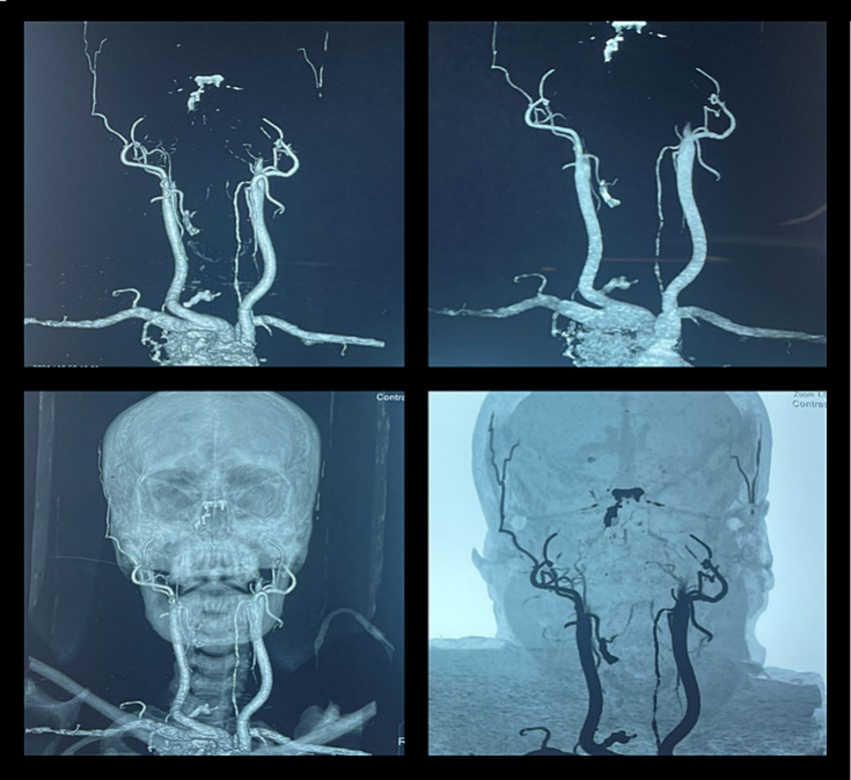
CT angiography in brain death. A 46-year-old woman was brought to our emergency department after a fatal motor vehicle accident. Her Glasgow Coma Scale (GCS) score was three on arrival. She had bradycardia and hypotension. The CT scan of the head showed a massive subdural hematoma with mass effect and compression of the brainstem with signs of herniation. She was intubated to secure the airway, and ICP was monitored by a subdural electrode. The raised ICP remained refractory to standard medical therapy, and her neurologic status progressively deteriorated and remained unresponsive. She further developed fixed dilated pupils, and her clinical findings were suggestive of brain death. The CT angiography showed non-opacification of the intracranial arteries beyond the internal carotid artery, suggestive of brain death.

Several studies have also demonstrated the use of CTA for the diagnosis of brain death (31, 32). The majority of these can be applied to individuals who are heavily sedated, and their utility is predictive rather than confirming (32). When a patient has clinical ambiguity about their diagnosis, CTA can provide some evidence to support the diagnosis of brain death by indirectly demonstrating the absence of contrast opacification of cerebral arteries (Figure 6) (32). While the specificity of CTA for diagnosing brain death is described as 100%, sensitivity ranges from 52 to 97% (33, 34).

MRA has been reported to carry a high sensitivity (93–100%) and specificity (100%), probably related to the additional, reliable, and detailed information about cerebral parenchymal changes (35, 36). MRA is rarely performed for the diagnosis of brain death as it is time-consuming, especially in critically ill patients. Furthermore, the commonly used “time-of-flight” sequences are not reliable due to their poor sensitivity to slow flow.

While interpreting the results of CTA, DSA, or MRA, clinicians should be cautious of the false-positive results in patients with hypotension and false-negative results in patients who have undergone decompressive craniectomy (22).

Table 1 summarizes the reported accuracy parameters of various ancillary tests presented in this review. We wish to reiterate that brain death remains a clinical diagnosis and essentially requires clinical demonstration of coma, absent brainstem reflexes, and apnea. There should be no evidence of arousal or awareness in response to maximal external stimuli that include noxious, visual, auditory and tactile stimulation. The pupils should be fixed in mid-size or dilated and non-reactive to light. Other findings include absent oculocephalic, oculovestibular reflexes, absent corneal reflexes, in addition to absent gag and cough reflexes.

**Table 1:** Salient characteristics and reported accuracy of various ancillary tests.

Test	Main diagnostic criteria	Sensitivity (%)	Specificity (%)	Comment
SPECT (15)	Absence of intracranial radionuclide uptake	88.4	100	Limited availability
DSA (16, 37)	No contrast opacification in intracranial vessels	100	100	Invasive; patient needs to be transferred to angiography suite
TCD (22)	Oscillating flow or small systolic spikes in intracranial arteries	90	98	Bedside; rapid; can be repeated easily; serial evaluation improved diagnostic accuracy
EEG (24, 37)	No detectable electrical activity (≥2 μV) over a 30-min period	53–80	97	Bedside; non-invasive; affected by sedation
SSEP (38)	No electrical transmission through brainstem and cerebrum along with intact signal in brachial plexus and spinal cord	100	78	Should be used in conjunction with EEG; less affected by sedation
CT brain	Extensive parenchymal damage	NA	NA	Shows only parenchymal damage; does not show brain function; should be interpreted along with CTA and other ancillary tests
MRI brain	Extensive parenchymal damage	NA	NA	Shows parenchymal damage with higher sensitivity; no information about brain function; should be interpreted with MRA and other ancillary tests
CTA brain (33, 39–41)	No opacification of intracranial arteries and deep veins	52–97	100	Widely available; quick; limited consensus on diagnostic criteria; not validated
MRA brain (28, 34, 35, 42)	No visualization of intracranial arteries	93–100	100	More time-consuming than CTA; widely available; not validated

CT, computerized tomography; CTA, CT angiography; DSA, digital subtraction angiography; EEG, electroencephalography; MRI, magnetic resonance imaging; MRA, MR angiography; NA, not applicable; SPECT, single photon emission computerized tomography; SSEP, somatosensory evoked potentials; TCD, transcranial Doppler.

## Conclusion

This review provides a concise and practical overview of various ancillary tests performed for the diagnosis of brain death. We have presented the test performance and utility of various ancillary tests for helping the diagnosis of brain death in adults in routine clinical practice. Our review provides the principles and methods of interpretation of various ancillary tests, along with respective clinical examples. The principles of performing and interpreting these ancillary tests do not vary according to country or region. Ancillary tests should be used to support the diagnosis of brain death only if a proper clinical examination cannot be completed. Multi-modal ancillary tests may be more useful. Various ancillary tests may be employed according to their availability at individual centers, underlying medical conditions as well as clinicians’ preference to support the diagnosis of brain death. However, we strongly believe that, if feasible, the ancillary tests may be repeated after a reasonable time has elapsed and various confounders have been corrected. While the ancillary tests may not be used for diagnosing brain death, such tests may guide the intensivists to optimize the timing of performing the necessary clinical examinations required to diagnose brain death.

## References

[1] Mollaret P Goulon M. Le coma dépassé. Rev Neurol. (1959) 101:3–15. 14423403

[2] Ad Hoc Committee. A definition of irreversible coma: report of the Ad Hoc Committee of the Harvard Medical School to Examine the Definition of Brain Death. JAMA. (1968) 205:337–40. doi: 10.1001/jama.1968.03140320031009 5694976

[3] Greer DM Shemie SD Lewis A Torrance S Varelas P Goldenberg FD. Determination of brain death/death by neurological criteria: the World Brain Death Project. JAMA. (2020) 324:1078–97. doi: 10.1001/jama.2020.11586, PMID: 32761206

[4] Spears W Mian A Greer D. Brain death: a clinical overview. J Intensive Care. (2022) 10:16. doi: 10.1186/s40560-022-00609-4, PMID: 35292111 PMC8925092

[5] Lewis A Kirschen MP Greer D. The 2023 AAN/AAP/CNS/SCCM pediatric and adult brain death/death by neurologic criteria consensus practice guideline: a comparison with the 2010 and 2011 guidelines. Neurol Clin Pract. (2023) 13:e200189. doi: 10.1212/CPJ.0000000000200189, PMID: 37829552 PMC10567121

[6] Lewis A Liebman J Kreiger-Benson E Kumpfbeck A Bakkar A Shemie SD. Ancillary testing for determination of death by neurologic criteria around the world. Neurocrit Care. (2021) 34:473–84. doi: 10.1007/s12028-020-01039-6, PMID: 32648194

[7] Lewis A Adams N Varelas P Greer D Caplan A. Organ support after death by neurologic criteria: results of a survey of US neurologists. Neurology. (2016) 87:827–34. doi: 10.1212/WNL.0000000000003008, PMID: 27449064

[8] Shemie SD Hornby L Baker A Teitelbaum J Torrance S Young K. International guideline development for the determination of death. Intensive Care Med. (2014) 40:788–97. doi: 10.1007/s00134-014-3242-7, PMID: 24664151 PMC4028548

[9] Plourde G Neves Briard J Shemie SD Shankar J Chassé M. Flow is not perfusion, and perfusion is not function: ancillary testing in brain death. Can J Anaesth. (2021) 68:953–61. doi: 10.1007/s12630-021-01988-2, PMID: 33942244 PMC8175303

[10] Neves Briard J Nitulescu R Lemoine É Titova P McIntyre L English SW. Diagnostic accuracy of ancillary tests for death by neurologic criteria: a systematic review and meta-analysis. Can J Anaesth. (2023) 70:736–48. doi: 10.1007/s12630-023-02426-1, PMID: 37155120 PMC10202988

[11] McKinnon NK Maratta C Zuckier LS Boyd JG Chassé M Hornby L. Ancillary investigations for death determination in infants and children: a systematic review and meta-analysis. Can J Anaesth. (2023) 70:749–70. doi: 10.1007/s12630-023-02418-1, PMID: 37131035 PMC10203011

[12] Drake M Bernard A Hessel E. Brain death. Surg Clin North Am. (2017) 97:1255–73. doi: 10.1016/j.suc.2017.07.001, PMID: 29132508

[13] Gonçalves FG Barra FR de Lima Matos W Young CL do Amaral LLF delCarpio-O’Donovan R. Signs in neuroradiology: part 1. Radiol Bras. (2011) 44:123–8. doi: 10.1590/S0100-39842011000200013

[14] Huang AH. The hot nose sign. Radiology. (2005) 235:216–7. doi: 10.1148/radiol.2351030537, PMID: 15798171

[15] Joffe AR Lequier L Cave D. Specificity of radionuclide brain blood flow testing in brain death: case report and review. J Intensive Care Med. (2010) 25:53–64. doi: 10.1177/0885066609355388, PMID: 20095080

[16] Braum M Ducrocq X Huot JC Audibert G Anxionnat R Picard L. Intravenous angiography in brain death: report of 140 patients. Neuroradiology. (1997) 39:400–5. doi: 10.1007/s002340050432, PMID: 9225317

[17] Wijdicks EFM Varelas PN Gronseth GS Greer DM American Academy of Neurology. Evidence-based guideline update: determining brain death in adults: report of the Quality Standards Subcommittee of the American Academy of Neurology. Neurology. (2010) 74:1911–8. doi: 10.1212/WNL.0b013e3181e242a8, PMID: 20530327

[18] Savard M Turgeon AF Gariépy JL Trottier F Langevin S. Selective 4 vessels angiography in brain death: a retrospective study. Can J Neurol Sci. (2010) 37:492–7. doi: 10.1017/S0317167100010520, PMID: 20724258

[19] Gastala J Fattal D Kirby PA Capizzano AA Sato Y Moritani T. Brain death: radiologic signs of a non-radiologic diagnosis. Clin Neurol Neurosurg. (2019) 185:105465. doi: 10.1016/j.clineuro.2019.105465, PMID: 31472395

[20] Rizvi T Batchala P Mukherjee S. Brain death: diagnosis and imaging techniques. Semin Ultrasound CT MR. (2018) 39:515–29. doi: 10.1053/j.sult.2018.01.006, PMID: 30244764

[21] Alvarez LA Lipton RB Hirschfeld A Salamon O Lantos G. Brain death determination by angiography in the setting of a skull defect. Arch Neurol. (1988) 45:225–7. doi: 10.1001/archneur.1988.00520260117031, PMID: 3341939

[22] Chang JJ Tsivgoulis G Katsanos AH Malkoff MD Alexandrov AV. Diagnostic accuracy of transcranial Doppler for brain death confirmation: systematic review and meta-analysis. AJNR Am J Neuroradiol. (2016) 37:408–14. doi: 10.3174/ajnr.A4548, PMID: 26514611 PMC7960140

[23] Ducrocq X Hassler W Moritake K Newell DW von Reutern GM Shiogai T. Consensus opinion on diagnosis of cerebral circulatory arrest using Doppler-sonography: Task Force Group on cerebral death of the Neurosonology Research Group of the World Federation of Neurology. J Neurol Sci. (1998) 159:145–50. doi: 10.1016/S0022-510X(98)00158-0, PMID: 9741398

[24] Grigg MM Kelly MA Celesia GG Ghobrial MW Ross ER. Electroencephalographic activity after brain death. Arch Neurol. (1987) 44:948–54. doi: 10.1001/archneur.1987.00520210048018, PMID: 3619714

[25] Stecker MM Sabau D Sullivan L das RR Selioutski O Drislane FW. American Clinical Neurophysiology Society guideline 6: minimum technical standards for EEG recording in suspected cerebral death. J Clin Neurophysiol. (2016) 33:324–7. doi: 10.1097/WNP.0000000000000322, PMID: 27482789

[26] Aminoff MJ. The use of somatosensory evoked potentials in the evaluation of the central nervous system. Neurol Clin. (1988) 6:809–23. doi: 10.1016/S0733-8619(18)30844-2 3070340

[27] Ramachandran S Venkatesh H Foley RW. How should we use imaging in the determination of brainstem death? BJR Open. (2018) 1:20180013. doi: 10.1259/bjro.20180013, PMID: 33178909 PMC7592410

[28] Sohn CH Lee HP Park JB Chang HW Kim E Kim E. Imaging findings of brain death on 3-tesla MRI. Korean J Radiol. (2012) 13:541–9. doi: 10.3348/kjr.2012.13.5.541, PMID: 22977320 PMC3435850

[29] Orrison WW Jr Champlin AM Kesterson OL Hartshorne MF King JN. MR “hot nose sign” and “intravascular enhancement sign” in brain death. AJNR Am J Neuroradiol. (1994) 15:913–6. PMID: 8059660 PMC8332192

[30] Taylor T Dineen RA Gardiner DC Buss CH Howatson A Pace NL. Computed tomography (CT) angiography for confirmation of the clinical diagnosis of brain death. Cochrane Database Syst Rev. (2014) 2018:CD009694. doi: 10.1002/14651858.CD009694.pub2, PMID: 24683063 PMC6517290

[31] Heran MK Heran NS Shemie SD. A review of ancillary tests in evaluating brain death. Can J Neurol Sci. (2008) 35:409–19. doi: 10.1017/S0317167100009069, PMID: 18973057

[32] Sawicki M Bohatyrewicz R Safranow K Walecka A Walecki J Rowinski O. Computed tomographic angiography criteria in the diagnosis of brain death: comparison of sensitivity and interobserver reliability of different evaluation scales. Neuroradiology. (2014) 56:609–20. doi: 10.1007/s00234-014-1364-9, PMID: 24801451 PMC4125746

[33] Garrett MP Williamson RW Bohl MA Bird CR Theodore N. Computed tomography angiography as a confirmatory test for the diagnosis of brain death. J Neurosurg. (2018) 128:639–44. doi: 10.3171/2016.10.JNS161042, PMID: 28304181

[34] Karantanas AH Hadjigeorgiou GM Paterakis K Sfiras D Komnos A. Contribution of MRI and MR angiography in early diagnosis of brain death. Eur Radiol. (2002) 12:2710–6. doi: 10.1007/s00330-002-1336-z, PMID: 12386761

[35] Luchtmann M Beuing O Skalej M Kohl J Serowy S Bernarding J. Gadolinium-enhanced magnetic resonance angiography in brain death. Sci Rep. (2014) 4:3659. doi: 10.1038/srep03659, PMID: 24413880 PMC3888970

[36] Paolin A Manuali A Di Paola F Boccaletto F Caputo P Zanata R. Reliability in diagnosis of brain death. Intensive Care Med. (1995) 21:657–62. doi: 10.1007/BF01711544, PMID: 8522670

[37] Su Y Yang Q Liu G Zhang Y Ye H Gao D. Diagnosis of brain death: confirmatory tests after clinical test. Chin Med J. (2014) 127:1272–7. doi: 10.3760/cma.j.issn.0366-6999.20133013, PMID: 24709179

[38] Combes JC Chomel A Ricolfi F d’Athis P Freysz M. Reliability of computed tomographic angiography in the diagnosis of brain death. Transplant Proc. (2007) 39:16–20. doi: 10.1016/j.transproceed.2006.10.204, PMID: 17275466

[39] Şahin H Pekcevik Y. CT angiography as a confirmatory test in diagnosis of brain death: comparison between three scoring systems. Diagn Interv Radiol. (2015) 21:177–83. doi: 10.5152/dir.2014.14241, PMID: 25698093 PMC4463321

[40] Mac Donald D Stewart-Perrin B Shankar JJS. The role of neuroimaging in the determination of brain death. J Neuroimaging. (2018) 28:374–9. doi: 10.1111/jon.12516, PMID: 29749664 PMC6055878

[41] Ishii K Onuma T Kinoshita T Shiina G Kameyama M Shimosegawa Y. Brain death: MR and MR angiography. AJNR Am J Neuroradiol. (1996) 17:731–5. PMID: 8730194 PMC8337280

